# Regulation of Autophagy-Related Protein and Cell Differentiation by High Mobility Group Box 1 Protein in Adipocytes

**DOI:** 10.1155/2016/1936386

**Published:** 2016-10-24

**Authors:** Huanhuan Feng, Lili Yu, Guojun Zhang, Guoyan Liu, Can Yang, Hui Wang, Xiangfeng Song

**Affiliations:** ^1^School of Basic Medical Sciences, Xinxiang Medical University, Xinxiang, Henan Province, China; ^2^Henan Collaborative Innovation Center of Molecular Diagnosis and Laboratory Medicine, Xinxiang Medical University, Xinxiang, Henan Province, China

## Abstract

High mobility group box 1 protein (HMGB1) is a molecule related to the development of inflammation. Autophagy is vital to maintain cellular homeostasis and protect against inflammation of adipocyte injury. Our recent work focused on the relationship of HMGB1 and autophagy in 3T3-L1 cells.* In vivo* experimental results showed that, compared with the normal-diet group, the high-fat diet mice displayed an increase in adipocyte size in the epididymal adipose tissues. The expression levels of HMGB1 and LC3II also increased in epididymal adipose tissues in high-fat diet group compared to the normal-diet mice. The* in vitro* results indicated that HMGB1 protein treatment increased LC3II formation in 3T3-L1 preadipocytes in contrast to that in the control group. Furthermore, LC3II formation was inhibited through HMGB1 knockdown by siRNA. Treatment with the HMGB1 protein enhanced LC3II expression after 2 and 4 days but decreased the expression after 8 and 10 days among various differentiation stages of adipocytes. By contrast, FABP4 expression decreased on the fourth day and increased on the eighth day. Hence, the HMGB1 protein modulated autophagy-related proteins and lipid-metabolism-related genes in adipocytes and could be a new target for treatment of obesity and related metabolic diseases.

## 1. Introduction

In the past 30 years, the global prevalence of obesity has increased among all age groups. Obesity leads not only to an increase in adipose tissue mass but also to the infiltration of proinflammatory cells and secretion of inflammatory cytokines [1, 2]. Therefore, obesity is characterized by low-grade inflammation in local and systemic sites as demonstrated by robust secretion of proinflammatory cytokines, including IL-6, as well as active recruitment of leukocytes [3]. Substantial evidence supports the hypothesis indicating that inflammation may contribute to insulin resistance, which further induces a series of diseases such as diabetes, hypertension, fatty liver disease, and coronary heart disease, thereby threatening human health [4, 5]. However, the mechanism underlying inflammation remains unclear.

Autophagy includes three basic forms, namely, macroautophagy, microautophagy, and chaperone-mediated autophagy (CMA) [6]. Macroautophagy (henceforth termed autophagy) is a lysosomal degradation pathway, which can degrade the organelles, longevity protein, and lipid drops and thus provide energy for the body [7, 8]. When the body faces various pressures caused by acute stress, autophagy plays a key role in maintaining the stability of the internal environment, particularly in regulating apoptosis and resisting the invasion of pathogenic microorganisms [9]. Self-renew, repair, and differentiation of cells are important for metabolism and maintenance of energy balance. Studies have shown that autophagic dysfunction is closely related to metabolic disorders, such as insulin resistance, diabetes, obesity, and osteoporosis [10].

High mobility group box 1 protein (HMGB1) is a nonhistone nuclear factor and a highly conserved protein. HMGB1 can bind to chromosomal DNA to adjust the refactoring of chromatin [11, 12]. HMGB1 is abundant in the vast majority of mammalian cells [13] and plays a key role as a signal molecule extracellularly [14]. HMGB1 can be passively released from necrotic cells or actively secreted from inflammatory cells [15, 16]. Aseptic injury to cells increases the level of HMGB1 in serum and tissues [17]. As such, HMGB1 is associated with low-grade inflammation diseases, such as obesity and type 2 diabetes [18]. Some research found that HMGB1 interacted with autophagy through its different receptors, outside the cells by receptor of advanced glycation end products (RAGE), within the nucleus through heat shock protein beta-1 (HSPB1), and within the cytoplasm through BECN1 [19]. These findings suggested that HMGB1 was involved in the process of autophagy. However, little is known about how HMGB1, autophagy, and adipocytes interact to regulate adipocyte development and differentiation. The present research mainly focused on the effects of HMGB1 on autophagy and cell differentiation in adipocytes.

## 2. Materials and Methods

### 2.1. Reagents

Antibodies were obtained from the following sources: HMGB1 and GAPDH from Abcam, LC3 from Cell Signaling, and p62 from Proteintech Group. Secondary antibodies against rabbit or mouse were bought from Beyotime. The following reagents were purchased from Sigma: 1-methyl-3-isobutylxanthine, dexamethasone, insulin, Oil-Red-O dye, and hematoxylin and eosin. The recombinant HMGB1 protein was obtained from Sino Biological. The negative control siRNA and siRNA HMGB1 were purchased from Invitrogen. TRIzol reagent and SuperScript III Reverse Transcriptase were also purchased from Invitrogen. SYBR® Select Master Mix was obtained from ABI.

### 2.2. Animals and Diet

C57BL/6 mice were purchased from Vital River Laboratory Animal Technology Co., Ltd., in Beijing. Rearing environment indoor temperature was controlled at 20°C to 25°C, relative humidity within 40% to 60%, lights 12 h every day, along with free drinking water in the cage. Six-week-old male mice were randomly divided into two groups, namely, the normal-diet (ND) group and high-fat diet (HFD) group, with 10 mice in each group. Mice were fed correspondingly with standard chow (10% kcal in fat) or HFD (45% kcal in fat) for 16 weeks. Mice's epididymal adipose tissue was extracted for the experiments.

### 2.3. Hematoxylin and Eosin Staining

Mouse epididymal adipose tissue samples were fixed in 4% paraformaldehyde and paraffin-embedded, then cut into 4 *μ*m thick sections, and deparaffinized in xylene and rehydrated in a descending ethanol series. The sections were stained with hematoxylin and eosin using standard pathologic procedures. Finally, photomicrographs of all sections were taken by Leica DM500 microscope.

### 2.4. Adipocyte Differentiation and Treatment of Recombinant HMGB1 Protein

3T3-L1 preadipocytes were maintained and cultured in Dulbecco's Modified Eagle's Medium (DMEM) with existing 10% heat inactivated fetal bovine serum and 1% penicillin/streptomycin at 37°C in 5% CO_2_. 3T3-L1 preadipocytes were first cultured in a medium supplemented with 10 *μ*g/mL insulin, 1 *μ*M dexamethasone, and 0.5 mM 1-methyl-3-isobutylxanthine for 2 days. The medium was then replaced by a medium containing only insulin. After 2 days, this maintenance medium was discarded. Differentiated adipocytes were cultured in DMEM containing 10% fetal bovine serum [1, 20].

To invest the effects of HMGB1 protein on autophagy in various differentiation periods of 3T3-L1 adipocytes, the adipocytes were treated with HMGB1 protein 24 h before differentiation. In detail, the 3T3-L1 preadipocytes were induced at 0, 2, 4, 6, 8, and 10 days, and the 0.2 *μ*g/mL recombinant HMGB1 proteins were added at days −1, 1, 3, 5, 7, and 9 and removed at days 0, 2, 4, 6, 8, and 10.

### 2.5. Cell Transfection

3T3-L1 preadipocytes were grown to approximately 50% to 70% confluence in a six-well plate and then transfected with 75 pmol HMGB1 siRNA and control siRNA by using Lipofectamine 2000 in accordance with the manufacturer's instructions. After transfection for 6 h, the medium was replaced with a normal medium.

### 2.6. Oil-Red-O Staining

Differentiated adipocytes were stained by Oil-Red-O, as follows: 0.5 g Oil-Red-O powder was dissolved in 100 mL of isopropanol overnight. The solution was filtered and stored in a brown bottle at 4°C. After the medium was discarded, cells were fixed with 4% formaldehyde for 2 h at room temperature. Cells were then washed with PBS and stained with the Oil-Red-O dye solution for 1 h. Subsequently, cells were washed with PBS and treated with 60% isopropanol for 5 min. Finally, cells were washed and photographed under a microscope.

### 2.7. Western Blot Analysis

3T3-L1 cells were collected and lysed in ice-cold lysis buffer (5.0 mM Tris buffer, pH 7.4, 150 mM NaCl, 1% NP-40, 0.1% SDS, 0.5% deoxycholic acid, 1 mM EDTA, 1 mM PMSF, 2 *μ*g/*μ*L aprotinin, 2 *μ*g/*μ*L leupeptin, and 1 mM NaF) for 40 min and then subsequently boiled and quantified. After the samples were resolved by SDS-PAGE for 2 h, proteins were transferred to nitrocellulose membranes at 200 V for 90 min. Membranes were blocked in 5% nonfat milk for 2 h and then incubated with specific primary antibodies for 2 h or overnight at 4°C. After being washed with Tris-buffered saline with Tween (TBST) thrice, membranes were incubated with peroxidase-conjugated secondary antibodies for 1 h. Finally, membranes were washed with TBST and developed by enhanced chemiluminescence. Blots were quantified using Amersham Imager 600 and analyzed with Image J.

### 2.8. Quantitative PCR

Total RNA was purified using TRIzol reagent and converted into cDNA by SuperScript III Reverse Transcriptase. The sequences of primers for PCR amplification were as follows: GAPDH: forward, 5′-AGGTCGGTGTGAACGGATTTG-3′; reverse, 5′-TGTAGACCATGTAGTTGAGGTCA-3′ [21]; PPAR-*γ*: forward, 5′-TTTTCAAGGGTGCCAGTTTC-3′; reverse, 5′-TCTGTGACGATCTGCCTGAG-3′; FABP4: forward, 5′-TCCAGTGAAAACTTTGATGATTAT-3′; reverse, 5′-ACGCATTCCACCACCAGTTTATCA-3′ [22]. Quantitative PCR analysis was performed using SYBR Select Master Mix in 7500 Fast Real-Time PCR System. The thermal profile for real-time PCR was 50°C for 2 min, 95°C for 10 min, followed by 40 cycles of 95°C for 15 s and 60°C for 1 min, and then 95°C for 30 s and 60°C for 15 s. Relative expression was calculated using the 2^−ΔΔCT^ method. All samples were normalized to GAPDH.

### 2.9. Enzyme-Linked Immunosorbent Assay (ELISA)

The level of peripheral blood HMGB1 was detected in mice at 16 weeks in accordance with the ELISA kit's protocol. The spectrophotometry (OD) value of the microplate reader was set at 450 nm wave length. The HMGB1 concentration in the testing samples can be calculated using the standard curve.

### 2.10. Statistics

Data are presented as means ± SEM. Significance was assessed by Student's *t*-test between individual comparisons. One-way analysis of variance with Bonferroni's corrections was used for multiple comparisons. Calculations were performed using SPSS version 19.0 statistic software. Differences were considered statistically significant at ^*∗*^ *P* < 0.05.

## 3. Results

### 3.1. Effects of HFD on Body Weight and Epididymal Adipose Tissue

**Figure 1:**
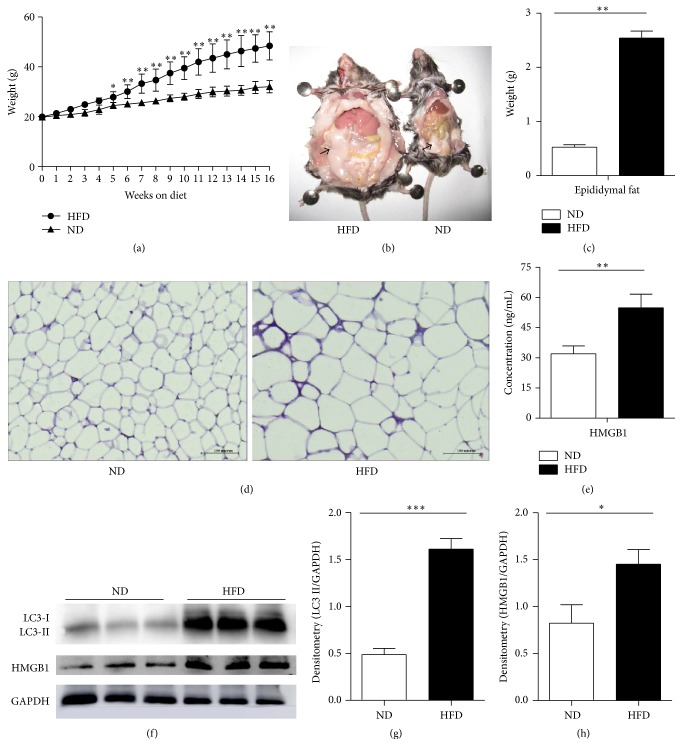
Effects of HFD on HMGB1 and LC3. After male mice were fed with HFD and ND for 16 weeks, serum was separated, and the epididymal adipose tissue was extracted. (a) The change of the body weight. (b) The epididymal adipose tissue, HFD (left) and ND (right). The black arrow pointed to epididymal adipose tissue. (c) The weight of epididymal adipose tissue. (d) HE staining of epididymal adipose tissue. (e) Levels of HMGB1 in peripheral blood were analyzed by ELISA. (f) The expression of autophagy protein LC3II and HMGB1 was analyzed by Western blot. (g-h) The protein levels were quantified through normalization with GAPDH, ^*∗*^ *P* < 0.05, ^*∗∗*^ *P* < 0.01, and ^*∗∗∗*^ *P* < 0.001 for comparisons between ND and HFD mouse epididymal adipose tissues.

To determine the changes in HMGB1 and autophagy protein LC3 in epididymal adipose tissues in obese mice, male C57BL/6 mice were fed with HFD for 16 weeks. The mean body weight for each group is shown in Figure 1(a). With the extension of time, the body weight significantly increased in the HFD group. After 16 weeks, the mean body weight of the HFD group was about 1.5 times that of the ND group. Moreover, the epididymal adipose tissue of the HFD group increased notably (Figures 1(b) and 1(c)). We also found that the adipocyte sizes in the HFD group were significantly larger than those in the ND group (Figure 1(d)).

### 3.2. HMGB1 and LC3 Were Upregulated in High-Fat Diet Mice

HMGB1 was assessed in epididymal adipose tissue and serum by Western blot and ELISA, respectively. Compared with the ND group, the HFD group showed that HMGB1 expression increased in both epididymal adipose tissues and peripheral blood of obese mice (Figures 1(e), 1(f), and 1(h)). Western blot was also used to detect the expression of autophagic protein LC3 in epididymal adipose tissues. The LC3II protein markedly increased, reaching 3.29 times that of the normal-diet groups (Figures 1(f) and 1(g)). These data suggested that HFD promoted HMGB1 production and activated autophagy in the epididymal adipose tissues in mice.

### 3.3. Exogenous HMGB1 Protein Promoted LC3II Formation and P62 Degradation in 3T3-L1 Preadipocytes

**Figure 2:**
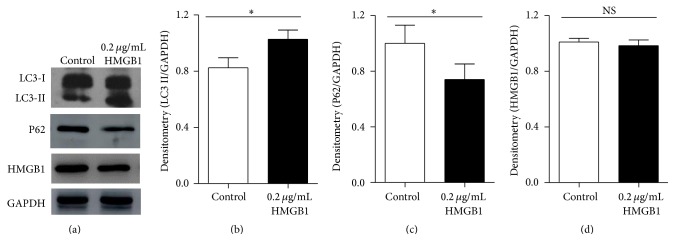
Exogenous HMGB1 activated autophagy. 3T3-L1 preadipocytes were treated with HMGB1 protein (0.2 *μ*g/mL) or PBS for 24 h accordingly. (a) Total protein was extracted, and LC3II, P62, and HMGB1 proteins were analyzed by western blot. (b–d) LC3II, P62, and HMGB1 protein were quantified and normalized to GAPDH, ^*∗*^ *P* < 0.05 versus the control. NS: not significant.

To determine the functional role of exogenous HMGB1 in autophagy, 3T3-L1 preadipocytes were treated with 0.2 *μ*g/mL recombinant HMGB1 protein for 24 h, which did not affect the normal cell proliferation [7] and HMGB1 protein expression in cells (Figures 2(a) and 2(d)). However, compared with the control group, the treatment group showed that the expression of autophagic protein LC3II increased (Figures 2(a) and 2(b)), whereas the P62 protein expression decreased significantly (Figures 2(a) and 2(c)).

### 3.4. Knockdown of HMGB1 Weakened LC3II Formation and P62 Degradation

**Figure 3:**
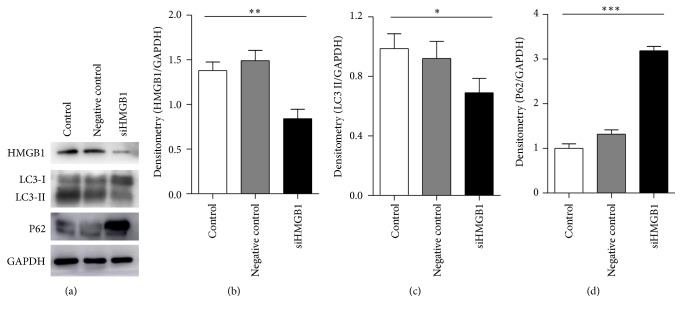
Autophagy was weakened by siRNA knockdown of HMGB1. HMGB1 siRNA or control siRNA was transfected into 3T3-L1 preadipocytes by lipo2000. (a) The expression levels of HMGB1, LC3, and P62 were examined by Western blot. (b–d) Quantification of protein levels normalized by GAPDH. ^*∗*^ *P* < 0.05, ^*∗∗*^ *P* < 0.01, and ^*∗∗∗*^ *P* < 0.001, versus the control.

We further studied the changes in autophagy when HMGB1 was downregulated by specific siRNA. Western blot analysis revealed that the knockdown of HMGB1 significantly reduced HMGB1 protein expression (Figures 3(a) and 3(b)). Furthermore, the LC3II protein decreased obviously, whereas the P62 protein increased markedly compared with the control group (Figures 3(c) and 3(d)).

### 3.5. Effects of HMGB1 Protein on Various Differentiation Periods of 3T3-L1 Adipocytes

**Figure 4:**
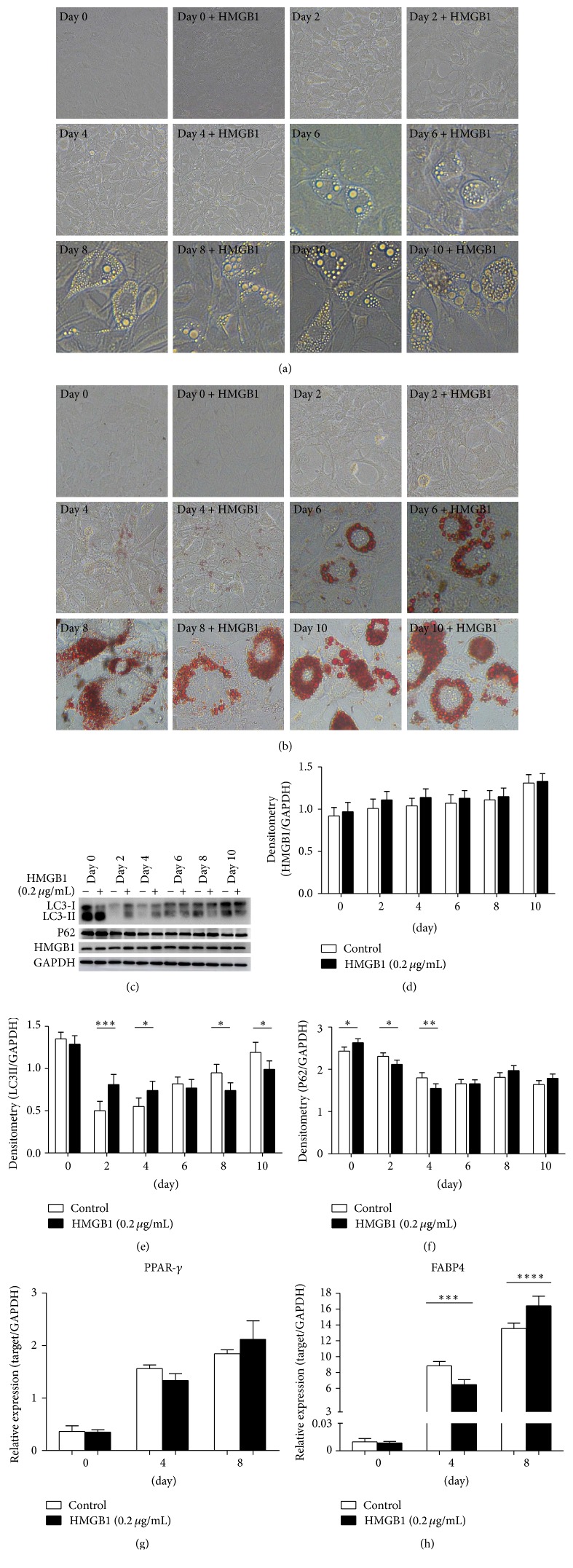
Effects of recombinant HMGB1 protein on autophagy in various differentiation periods of 3T3-L1 adipocytes. Differentiation of 3T3-L1 preadipocytes was induced at 0, 2, 4, 6, 8, and 10 days. The adipocytes were added with 0.2 *μ*g/mL recombinant HMGB1 protein 24 h before differentiation. (a) Pictures were taken with a bright-field microscopic images of differentiating cells. (b) Oil-Red-O staining (200 magnification). (c) The expression of proteins HMGB1, LC3, and P62 was detected by Western blot. (d–f) HMGB1, LC3, and P62 protein were quantified and normalized to GAPDH. (g-h) Levels of PPAR-*γ* and FABP4 were determined by quantitative PCR in differentiation at 0, 4, and 8 days, ^*∗*^ *P* < 0.05, ^*∗∗*^ *P* < 0.01, ^*∗∗∗*^ *P* < 0.001, and ^*∗∗∗∗*^ *P* < 0.0001 versus the day itself (control).

To further explore the effects of HMGB1 on autophagy in various differentiation periods of 3T3-L1 adipocytes, we added 0.2 *μ*g/mL HMGB1 protein in the differentiation stages of adipocytes for 24 h. Oil-Red-O staining was performed to detect intracellular lipid droplets. As shown in Figures 4(a) and 4(b), 3T3-L1 preadipocytes were successfully induced into the adipocytes in the control group. No significant difference was observed in the HMGB1 treatment group. Interestingly, with the differentiation of adipocytes, the LC3II protein expression increased gradually. However, after adding exogenous HMGB1 for 24 h, the LC3II expression increased in the differentiation on the second and fourth days but decreased in the differentiation on the eighth and tenth days compared with the control group (Figures 4(c) and 4(e)). On the contrary, the P62 protein expression decreased in the differentiation on the second and fourth days (Figures 4(c) and 4(f)). Finally, the expression of adipocyte differentiation related genes peroxisome proliferator-activated receptor- (PPAR) *γ* and FABP (fatty acid-binding protein) 4 was measured. No significant difference in PPAR-*γ* was found between the two groups (Figure 4(g)), but the FABP4 expression significantly decreased in 4 days and increased in 8 days (Figure 4(h)). These data indicated that the HMGB1 protein exerted different effects on autophagy and lipid metabolism in the early and late stages of adipocyte differentiation.

## 4. Discussion

The adipose tissue plays an important role in whole-body energy homeostasis. The two main types of adipose tissues are white and brown. White adipose tissue is primarily responsible for energy storage of the body [23], whereas brown adipose tissue is mainly responsible for energy degradation through uncoupling protein 1 (UCP1) [24]. Obesity is characterized by an excess of white adipose tissue [25] and leads to adipocyte dysfunction, thereby increasing the risk for insulin resistance, type 2 diabetes mellitus, and cardiovascular diseases [26, 27].

HMGB1 is produced as a damage-associated molecular pattern molecule (DAMP) by necrotic cells or activated immune cells [14]. Studies showed that HMGB1 protein expression increased in the adipose tissues of obese mice [28]. Our data also confirmed the increased expression of HMGB1 in the adipose tissues and peripheral blood of mice induced by HFD.

Autophagy is a process which passes intracytoplasmic ingredients to lysosomes for degradation and provides energy to the body [29]. Autophagy not only is very important in the maintenance of cellular homeostasis but also participates in the metabolism of lipid droplets and lipogenesis. Research showed that, in both* in vivo* and* in vitro* experiments, the inhibition of autophagy increases lipid storage. This finding may link to the reduction of the degradation of triacylglycerol. In addition, knockdown of autophagy genes Atg5 and Atg7 in adipocytes can improve insulin sensitivity and relieve obesity [30]. Therefore, autophagy played an important role in regulating obesity-related metabolic disorders [31]. The suppression of autophagy also reduced the expression of adipogenesis-related genes, such as PPAR-*γ* and C/EBPs. Previous studies proved that the knockdown of Atg7 decreased TG accumulation in 3T3-L1 preadipocytes, generated obese mice with decreased weight and white adipose mass, and then enhanced insulin sensitivity. These findings suggest that autophagy regulated lipid metabolism.

Some research found that HMGB1 activated an autophagic response to oxidative stress [32], and the loss of HMGB1 inhibited autophagy. When HMGB1 decreased, the autophagy weakened. We observed the change of autophagy in 3T3-L1 preadipocytes when HMGB1 was disturbed by specific small interfering RNA. Our results showed that autophagy was weakened when the expression of HMGB1 was suppressed in 3T3-L1 preadipocytes. HMGB1 also played a vital role in regulating autophagy in response to metabolic stress and oxidative damage [33]. However, little is known about the connections between the HMGB1 protein and the change of autophagy in adipose tissues of mice. Our results displayed that HMGB1 increased along with autophagy activated in adipose tissue of obese mice induced by HFD. This finding implied that activated autophagy may be a protective mechanism under cell stress state induced by HFD.

To gain insights into the effects of HMGB1 on autophagy in adipocytes,* in vitro* studies were performed in adipocytes. 3T3-L1 preadipocytes were first stimulated with the recombinant HMGB1 protein to observe the expression of autophagy-related protein LC3 (microtubule-associated protein 1A/1B-light chain 3) and P62. A cytosolic form of LC3 (LC3I) is conjugated to phosphatidylethanolamine to form LC3-phosphatidylethanolamine conjugate (LC3II), which is recruited to autophagosomal membranes [34, 35]. Thus, the formation of LC3II appears to be correlated with the induction of autophagy [36]. P62 serves as a link between LC3 and ubiquitinated substrates, and the inhibition of autophagy correlates with the increased levels of p62. Our results showed that LC3II increased and P62 decreased when cells were treated with recombinant HMGB1 protein. This finding suggests that exogenous HMGB1 induced autophagy in 3T3-L1 preadipocytes. In addition, as small interfering RNA knocked down the expression of HMGB1, autophagy appeared to be weakened. These data indicated that HMGB1 played an important role in regulating the autophagy in 3T3-L1 preadipocytes.

We further examined whether HMGB1 proteins were related to adipocyte differentiation and autophagy. 3T3-L1 adipocytes were cotreated with HMGB1 protein and adipocyte differentiation-inducing agent. We observed HMGB1 activated autophagy in the early differentiation within 2 and 4 days but weakened autophagy in differentiation after 8 and 10 days. Presumably, the activation of autophagy resisted external stress in the early differentiation stage but was unable to resist this constant pressure in the late stage of differentiation. These results demonstrated that HMGB1 played a different role in the differentiation of 3T3-L1 adipocytes. Several transcription factors, such as PPAR-*γ*, are involved in the differentiation of preadipocytes into mature adipocytes [37]. FABP4, a lipid chaperone, is expressed in adipocytes and plays an important role in the regulation of insulin sensitivity [38]. Our results showed that FABP4 increased within 4 days but inversed within 8 days. This finding was consistent with the expression of LC3, which suggested that HMGB1 regulated autophagy and further altered lipid metabolism in adipocytes.

## 5. Conclusion

This study demonstrated that HFD induced HMGB1 protein and LC3II production in adipose tissues. HMGB1 can regulate autophagy and alter lipid metabolism in adipocytes. These results not only provided a new theoretical basis for HMGB1 and autophagy in adipocytes but also presented a new target for the treatment of obesity and its related metabolic diseases.
